# Time From Onset to Diagnosis of Alpha-Gal Syndrome

**DOI:** 10.1001/jamanetworkopen.2024.61729

**Published:** 2025-03-10

**Authors:** Caroline K. Maki, Eleanor F. Saunders, Marissa L. Taylor, Scott P. Commins, Lance A. Waller, Johanna S. Salzer

**Affiliations:** 1 Rickettsial Zoonoses Branch, Division of Vector Borne Diseases, Centers for Disease Control and Prevention, Atlanta, Georgia; 2 Oak Ridge Institute for Science Education, Oak Ridge, Tennessee; 3 Gangarosa Department of Environmental Health, Emory University Rollins School of Public Health, Atlanta, Georgia; 4 Institute for Global Health and Infectious Diseases, University of North Carolina at Chapel Hill; 5 Departments of Medicine & Pediatrics, Division of Allergy and Immunology, University of North Carolina at Chapel Hill; 6 Department of Biostatistics and Bioinformatics, Emory University Rollins School of Public Health, Atlanta, Georgia

## Abstract

This case series investigates trends in time from onset of alpha-gal syndrome to diagnosis among patients with disease onset from 1977 to 2019.

## Introduction

Alpha-gal syndrome (AGS) is an emerging, tick-borne allergy to the galactose-α-1,3-galactose (alpha-gal) carbohydrate, estimated to impact up to 450 000 individuals in the US.^1^ This molecule is found in mammalian meat, mammal-derived products, and certain pharmaceuticals. In the US, the distribution of AGS cases closely resembles the distribution of the lone star tick, *Amblyomma americanum*.^1^ Symptoms present 2 to 8 hours after consuming a product containing alpha-gal and can be life-threatening. A 2022 survey found that 42% of health care practitioners in the US had never heard of AGS and an additional 35% were “not too confident” in their ability to diagnose and manage the condition.^2^ Challenges in receiving timely care can reveal disparities among various patient populations eventually diagnosed. We analyzed a group of patients with AGS seeking care at an allergy clinic in North Carolina for factors that may have influenced their ability to receive a timely and accurate diagnosis. A comparison with a previous AGS cohort from the same allergy clinic was performed to understand diagnosis trends over time.^3^

## Methods

Data used in this case series were collected through a case-control study conducted by the Centers for Disease Control and Prevention’s Rickettsial Zoonoses Branch and the University of North Carolina at Chapel Hill (UNC) Allergy & Immunology Clinic. The study was approved by the UNC institutional review board, and participants provided written informed consent. Patients were recruited directly from the UNC Allergy and Immunology Clinic in Chapel Hill, and details of recruitment and demographic information are reported elsewhere.^4,5^ For the time-to-diagnosis variable, we assign 2009 as the earliest date of onset for patients reporting symptom onset earlier, given the formal recognition of AGS in the medical literature in 2009.^6^ However, for comparison with a previous study of UNC patients with AGS, symptom times prior to 2009 were included for consistency.^3^ All data were managed using R Studio 4.3.3 (R Project for Statistical Computing) and Microsoft Excel version 2402 from April to October 2024. Two-sided *P* < .05 was considered statistically significant. This study follows the reporting guideline for case series.

## Results

Of the 83 enrolled patients with AGS, 57 (69%) were residents of North Carolina; 73 (88%) self-reported White race and 46 (55%) reported female sex^4,5^; 72 (87%) responded to both the symptom duration survey questions and applicable demographic questions. For the quarter of patients with the earliest reported symptom onsets from 1977 to 2011 (n = 18; restricted for analysis to 2009-2011), the mean (SD) diagnosis time was 5.3 (3.7) years (Figure A). For the quarter of patients with the most recent symptom onsets, all in 2019 (n = 18), the mean (SD) diagnosis time was 28 (24) days, a 70-fold improvement from the earliest group. Additionally, we recorded a 3-year mean difference in diagnosis time between the highest and lowest reported education groups: 1.4 years for those with graduate degrees compared with 4.4 years for high school diploma holders (Figure B). No significant associations were found across other demographic variables.

**Figure.**
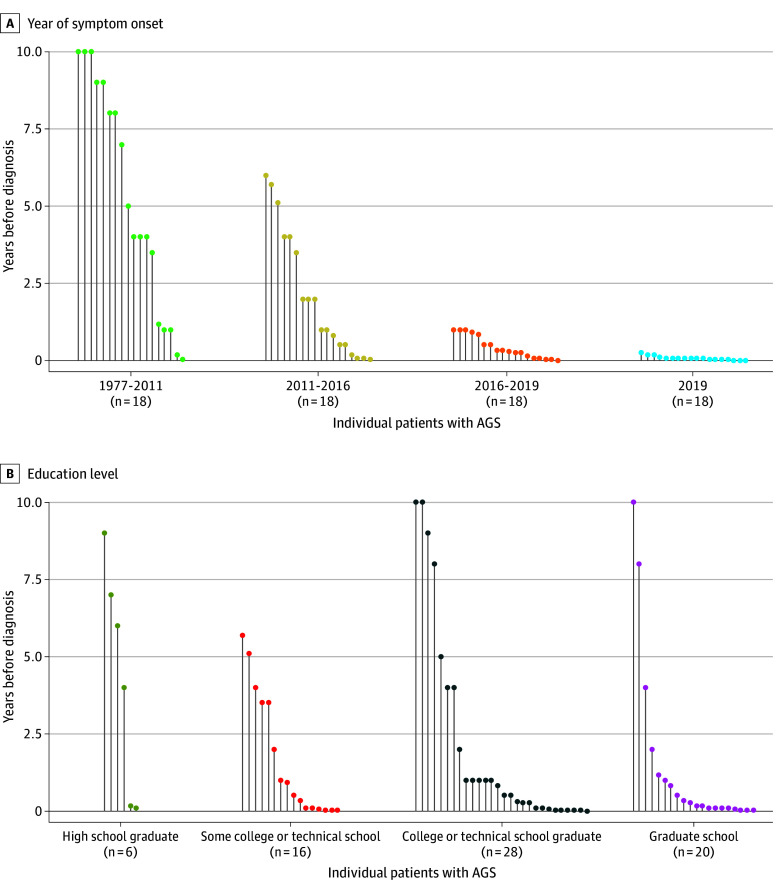
Diagnosis Time of Patients With AGS by First Symptom Onset and Education Level Pin plot of the time to diagnosis of patients in the case cohort. First year of symptom onset groups based on equal quartiles of 18 and labeled based on self-reported first year of onset (*P* < .001). Education groups were self-reported highest level of completed education (*P* = .50). Time in years does not include prior to 2009. Due to the division of data in 4 equal quartiles in panel A, the years 2011, 2016, and 2019 span multiple quartiles, though each observation is distinct.

A previous study analyzed the diagnosis time of 28 unique patients from the same clinic diagnosed prior to late 2015.^3^ Using the same methods, we found a significant increase in the proportion of patients diagnosed in less than a year and a significant decrease in those requiring more than 5 years when compared with the previous cohort (Table). The mean diagnosis time of diagnoses greater than 1 year was unchanged at 7.5 years.

**Table.** Count and Mean Time to Diagnosis of Patients With AGS^a^

Study	Time from AGS onset to diagnosis					
	<1 y		≥1 y^b^		≥5 y^b^	
	No. (%)	Mean (SD), y	No. (%)	Mean (SD), y	No. (%)	Mean (SD), y
Flaherty et al,^3^ 2017 (n = 28)^c^	6 (21.4)	NA	22 (78.6)	7.1 (NA)	9 (32.1)	NA
Maki et al, 2025 (n = 76)^c^	50 (65.7)	0.19 (0.23)	26 (34.3)	7.5 (7.9)	16 (21.1)	12.1 (8.4)

Abbreviations: AGS, alpha-gal syndrome; NA, not available.

^a^ Time to diagnosis of patients in the case cohort (n = 76) compared with patients within a previous University of North Carolina cohort of patients with AGS prior to late 2015 (n = 28). Displays count and percentage of patients with diagnoses less than 1 year, more than 1 year, and more than 5 years after onset. The mean diagnosis time of patients and the respective SD is included for both studies; however, insufficient data from Flaherty et al was accessible to calculate this variable for less than 1 year and greater than 5 years. Time in years includes years prior to 2009.

^b^ The variable for 1 year or more after onset also includes data from the variable for 5 years or more after onset.

^c^ The increase in diagnoses less than 1 year after onset and decrease in diagnoses 5 years or more after onset between Flaherty et al and Maki et al (current study) was significant (*P* = .01) under Pearson χ^2^ test.

## Discussion

In this cohort, the time between first symptom onset and confirmed AGS diagnosis has shortened 70-fold over time. Higher educational attainment was the only measured demographic factor associated with improved diagnosis time. The delay in diagnosis for those with lower levels of education highlights a clear gap in access to care, even among this unique cohort with eventual access to a medical specialist and AGS expert.

A limitation of our study is that it does not account for all disparities that may exist upstream of presenting to care. Underscoring such disparities in AGS diagnoses should continue in more representative datasets. As we learn more about the growing population of patients with AGS in the US, the disparities suggested by this study may become more apparent. Health care practitioners should have a well-rounded awareness of the factors that may influence patients’ abilities to receive timely AGS diagnoses to ensure the highest level of care and avoid undue physical, mental, and financial burden.

## Notes

Supplement.
Data Sharing Statement
