# Supplementary material for: Time From Onset to Diagnosis of Alpha-Gal Syndrome
Source: JAMA Netw Open. 2025 Mar 10;8(3):e2461729. doi: 10.1001/jamanetworkopen.2024.61729 (PMC11894482; doi:10.1001/jamanetworkopen.2024.61729)
Supplement: Supplement. — Data Sharing Statement [file jamanetwopen-e2461729-s001.pdf]

Data Sharing Statement

Maki. Time From Onset to Diagnosis of Alpha-Gal Syndrome. *JAMA Netw Open*. Published February 25, 2025. doi:10.1001/jamanetworkopen.2024.61729

Data
Data available: No
